# Increasing spectral DCM flexibility and speed by leveraging Julia’s ModelingToolkit and automated differentiation

**DOI:** 10.1162/IMAG.a.88

**Published:** 2025-07-24

**Authors:** David Hofmann, Anthony G. Chesebro, Chris Rackauckas, Lilianne R. Mujica-Parodi, Karl J. Friston, Alan Edelman, Helmut H. Strey

**Affiliations:** Laufer Center for Physical and Quantitative Biology, State University of New York at Stony Brook, Stony Brook, NY, United States; Computer Science and Artificial Intelligence Lab, Massachusetts Institute of Technology, Cambridge, MA, United States; Department of Biomedical Engineering, State University of New York at Stony Brook, Stony Brook, NY, United States; Athinoula A. Martinos Center for Biomedical Imaging, Massachusetts General Hospital and Harvard Medical School, Boston, MA, United States; Santa Fe Institute, Santa Fe, NM, United States; Wellcome Trust Centre for Neuroimaging, University College London, London, United Kingdom

**Keywords:** neural, circuit, causal, model, DCM, SPM, neuroimaging, LFP, fMRI, Julia, Neuroblox

## Abstract

Using neuroimaging and electrophysiological data to infer neural parameter estimations from theoretical circuits requires solving the inverse problem. Here, we provide a new Julia language package designed to i) compose complex dynamical models in a simple and modular way with ModelingToolkit.jl, ii) implement parameter fitting based on spectral dynamic causal modeling (sDCM) using the Laplace approximation, analogous to MATLAB implementation in SPM, and iii) leverage Julia’s unique strengths to increase accuracy and speed by employing Automatic Differentiation during the fitting procedure. To illustrate the utility of our flexible modular approach, we provide a method to improve correction for fMRI scanner field strengths (1.5T, 3T, 7T) when fitting models to real data.

## 1 Introduction

Answering fundamental questions in neuroscience usually requires abstracting from neuronal populations to circuits (K. J. Friston et al., 2014; Hahn et al., 2020; Lee et al., 2022; Stephan et al., 2008). Neuronal circuits form causal models of interactions between different regions of the brain. Estimating model parameters, such as effective connectivity between brain regions, further requires data fitting (K. J. Friston, 2011; K. J. Friston et al., 2014; Stephan et al., 2008). One powerful approach is Dynamic Causal Modeling (DCM), introduced as part of the Statistical Parametric Mapping (SPM) toolbox (K. J. Friston et al., 2014; Stephan et al., 2008). DCM provides methods and an associated pipeline for modeling interactions between neuronal populations at the cortical level, fitting models from local field potential (LFP) recordings (K. J. Friston et al., 2015; R. J. Moran, Jung, et al., 2011; R. J. Moran, Mallet, et al., 2011), electroencephalography (EEG) data (David et al., 2006), and functional MRI (fMRI) sequences (Cha et al., 2016; Rupprechter et al., 2020; Seghier & Friston, 2013; Sladky et al., 2015; Tik et al., 2018). Neuroscience applications of DCM have provided insights into both healthy (Bukhari et al., 2022; Hahn et al., 2020; Lee et al., 2022; Sladky et al., 2022) and impaired (Zarghami et al., 2023) neuronal functioning, in response to task-based (Hahn et al., 2020; Lee et al., 2022; Sladky et al., 2022) and resting-state (K. J. Friston et al., 2014; Zarghami et al., 2023) stimuli. Furthermore, the underlying mathematical approach of DCM has been extended to applications outside of neuroscience that benefit from causal inference (Parr et al., 2021).

DCM is a procedure used to infer model parameters from data and perform model comparison and selection (Novelli et al., 2024; Penny et al., 2004; Stephan et al., 2007; Zeidman et al., 2022). Models are defined to describe the underlying neuronal dynamics, typically at the meso- or macro-scale, as well as the measurement process (BOLD, EEG, LFP). A variational Bayes approach is used to estimate the posterior distribution of the parameters by optimizing the evidence lower bound (R. J. Moran, Jung, et al., 2011; Novelli et al., 2024; Zeidman et al., 2022). Finally, the evidence lower bound or negative free energy is used for model comparison (K. Friston et al., 2007; Razi et al., 2017). Users can include and exclude parameters, such as connection weights between regions by changing the prior distribution. Additionally, through model comparison, the user can judge which regions are most likely connected and whether they are excitatory or inhibitory. Over time, this approach has evolved and become more powerful, including adopting a computationally more efficient DCM algorithm in the spectral domain (K. J. Friston et al., 2014). Signals, assumed to be stationary, are transformed to the spectral domain by the computation of cross-spectral densities (CSD). This computation is performed by fitting an autoregressive (AR) model to the time series (in SPM the AR model is of fixed order 8). From the parameters of the AR model, one can analytically calculate the CSD. In our package, the fitting of the AR model is performed by standard maximum likelihood. Note that the standard procedure in SPM is a Bayesian fit defined in *spm_mar.m*.

The most current implementation of DCM, as part of the Statistical Parametric Mapping toolbox (SPM; available at fil.ion.ucl.ac.uk/spm/software/spm12/), has been widely and successfully applied to a variety of data modalities, cognitive functions, and disease processes (Hahn et al., 2020; Lee et al., 2022; Sladky et al., 2022; Zarghami et al., 2023). Here, we build on this achievement to extend its capabilities in two ways. First, limitations due to computation speed have been one of the practical roadblocks to the wide adoption of multi-scale computational neuroscience. DCM’s implementation in MATLAB introduces inherent computational barriers that become increasingly problematic as datasets scale to meet current reproducibility standards (Marek et al., 2022). For this reason, we chose to code our package in Julia, as part of the Neuroblox computational neuroscience platform (neuroblox.org), as Julia is a high-performance computing language that is known to show orders of magnitude speed improvements in ODE systems compared to both MATLAB and Python (Rackauckas, 2023). Second, we wanted to incorporate additional flexibility into the pipeline, ensuring easy access to choices for neural mass models, model assembly, transfer functions within and between scales and modalities, and parameter fitting procedures.

In this work, we provide a new implementation of spectral DCM in the Julia programming language (julialang.org), based on the ModelingToolkit package (Ma, Gowda, et al., 2021). We demonstrate that this implementation can offer results identical to the original SPM implementation at a fraction of the computation speed in an open-source language. Additionally, we constructed this implementation to allow for great user flexibility in model selection and specification, opening the door to new modeling techniques not achieved in prior implementations.

## 2 Theory

### 2.1 Spectral dynamic causal modeling

The distinctive feature of spectral DCM, as compared to other forms of DCM, is that it models the cross-spectral density of a signal. This implies the assumption of stationarity of the covariance matrix of the signals during measurement (Novelli et al., 2024). The usual continuous-time state-space formulation with a measurement function is assumed

$$\begin{array}{cc} \begin{array}{cc} \vec{\dot{x}} & =f\left(\vec{x};{\vec{\theta }}_{f}\right)+{\vec{\epsilon }}_{f}\\ \vec{y} & =g\left(\vec{x};{\vec{\theta }}_{g}\right)+{\vec{\epsilon }}_{g} \end{array} & \end{array}$$ (1)

For Eqns. 1, $\vec{y}$ denotes the measurement of the (hidden) dynamics $\vec{x}$ , which could, for example, be the neuronal dynamics and hemodynamic response. ${\vec{\epsilon }}_{f}$ and ${\vec{\epsilon }}_{g}$ denote intrinsic or endogenous noise and measurement noise, respectively. In spectral DCM, these are assumed to follow a power law distribution in frequency space. In fMRI measurements, the states x comprise not just the neuronal dynamics $\left({\vec{x}}_{n}\right)$ but also the hemodynamic response to the neuronal activity $\left({\vec{x}}_{h}\right)$. A further assumption made by spectral DCM is linearity of the hidden neuronal dynamics $f_{n}\left({\vec{x}}_{n};{\vec{\theta }}_{n}\right)={\vec{\theta }}_{n}{\vec{x}}_{n}$. The dynamics for the hemodynamic response $f_{h}\left({\vec{x}}_{h};{\vec{\theta }}_{h}\right)$ are expressed by the balloon model, and the measured signal is the blood oxygen level dependent (BOLD) denoted by $g\left({\vec{x}}_{h};{\vec{\theta }}_{g}\right)$. Both models are detailed in (Stephan et al., 2007), which is based on (Buxton et al., 2004). Here, we describe the model used in the code in more detail. The balloon model describes the dynamics of blood vessels in response to the neuronal signals. The cerebral blood flow u(t) is driven by the neuronal activity $x_{n}$ (for the sake of notational simplicity, we omit an index that denotes a specific region of the multidimensional vector ${\vec{x}}_{n}$ describing the neuronal activity of all regions. In what follows, all dynamic variables relate to a single region.) through the induction of a vasodilatory signal s(t) governed by the following dynamics:

$$\begin{array}{cc} \dot{s} & =x_{n}-\kappa s-\gamma \left(u-1\right) \end{array}$$ (2)

$$\dot{u}=s$$ (3)

where κ is the rate constant of the vasodilatory signal decay and γ is the rate constant for autoregulatory feedback by blood flow. Moreover, the change in volume v(t) due to the blood flow as well as the related changes of the blood oxygenation level q(t) are given, according to (Buxton et al., 2004), by

$$\begin{array}{c} \tau \dot{v}=u-v^{1/\alpha }\\ \tau \dot{q}=uE\left(u,E_{0}\right)\text{​}/E_{0}-v^{1/\alpha }q/v \end{array}$$ (4)

with $E\left(u,E_{0}\right)=1-{\left(1-E_{0}\right)}^{1/u}$ as a reasonable approximation across a wide range of conditions (Buxton & Frank, 1997; Stephan et al., 2007). Here, $E_{0}$ is the oxygen extraction fraction at rest, τ is the mean transit time at rest, and α is Grubb’s vessel stiffness exponent (Stephan et al., 2007). Finally, based on blood oxygenation q(t) and blood volume v(t) we compute the BOLD signal according to the following equation

$$\lambda \left(q,v\right)=V_{0}\left[k_{1}\left(1-q\right)+k_{2}\left(1-\frac{q}{v}\right)+k_{3}\left(1-v\right)\right]$$ (5)

with the following parameter definitions

$$\begin{array}{cc} \begin{array}{cc} \begin{array}{c} k_{1}=4.3\theta_{0}E_{0}T_{E}\\ k_{2}=\epsilon \;r_{0}E_{0}T_{E}\\ k_{3}=1-\epsilon \end{array} & \end{array} & \end{array}$$

where ϵ is the ratio of intra- and extravascular BOLD signal at rest, $\theta_{0}$ is the frequency offset at the outer surface of the magnetized vessel for fully deoxygenated blood, $E_{0}$ is the resting oxygen extraction fraction, $T_{E}$ is the echo time, and $r_{0}$ is the slope of the relation between the intravascular relaxation rate and oxygen saturation. The solution to the differential equations Eqns. 1 can be approximated by the first order of a Taylor expansion as

$$\begin{array}{cc} \vec{y}\left(t\right)=\kappa \left(\tau \right)*{\vec{\epsilon }}_{f}\left(t\right)+{\vec{\epsilon }}_{g}\left(t\right)\;\text{with}\;\kappa \left(\tau \right)=\nabla g\left(\vec{x}\right)\;\text{exp}\left(J_{f\left(\vec{x}\right)}\tau \right) & \end{array}$$ (6)

where $J_{f\left(\vec{x}\right)}$ denotes the Jacobian of f w.r.t. $\vec{x}$ in Eqn. 1. To find an expression for the cross-spectral density, we Fourier transform Eqn. 6

$$\tilde{\vec{y}}\left(\omega \right)=\tilde{\kappa }\left(\omega \right){\tilde{\vec{\epsilon }}}_{f}\left(\omega \right)+{\tilde{\vec{\epsilon }}}_{g}\left(\omega \right)$$ (7)

where $\tilde{\kappa }$, ${\tilde{\vec{\epsilon }}}_{f}$, and ${\tilde{\vec{\epsilon }}}_{g}$ denote the Fourier transforms of the convolution kernel and noises, respectively. The cross-spectral density is then

$$\begin{array}{cc} \begin{array}{cc} S_{y}\left(\vec{\theta }\right) & =\langle \tilde{\vec{y}}\left(\omega \right)\cdot \tilde{\vec{y}}{\left(\omega \right)}^{*}\rangle \\ & =\tilde{\kappa }\left(\omega \right)S_{\epsilon_{f}}\tilde{\kappa }{\left(\omega \right)}^{*}\;+S_{\epsilon_{g}} \end{array} & \end{array}$$ (8)

where * denotes the complex conjugate and $S_{\epsilon_{f}}$ and $S_{\epsilon_{g}}$ are the cross-spectral densities of the state noise and measurement noise, respectively. Note that these are diagonal matrices due to our assumption of uncorrelated noise. While the cross-spectrum of the measurement noise will have identical values on the diagonal, the state noise is composed of a global and a region-specific component, and thus diagonal elements can vary. This deviates from the presentation in (K. J. Friston et al., 2014) but is consistent with the implementation in SPM25. Note further that this derivation of the CSD implies that the underlying model is expanded at a fixed point solution in Eqn. 6, that is, the model cannot be in a limit cycle or another dynamical state (Bastos & Schoffelen, 2015; R. Moran et al., 2008).

We thus derived a linear approximation of the cross-spectral density $S_{y}\left(\vec{\theta }\right)$ that is based on the details of the time-continuous state space model given in Eqns. 1. Next, we would like to estimate the parameters of the model and the noise parameters and, therefore, need to define an optimization procedure. We follow the DCM approach, a Variational Bayes approach with Laplace approximation, termed Variational Laplace for brevity (Zeidman et al., 2022).

### 2.2 Variational Laplace

An inverse problem consists of estimating parameters from data. In a Bayesian setting, this implies computing the posterior distribution

$$p(\theta |y)=\frac{p(y|\theta )p\left(\theta \right)}{Z}$$ (9)

In the ideal setting, the true posterior distribution can analytically be computed from the likelihood p(y|θ) , the prior distribution p(θ), and the normalization Z, also called the model evidence or marginalized likelihood (Z=p(y)=∫p(y|θ)p(θ)dθ ) from the Bayes formula (Eqn. 9). However, posterior distributions typically do not have a closed-form solution. Instead, we have to either resort to sampling techniques (e.g., Markov Chain Monte Carlo) or compute an approximation to the posterior. Variational Bayes is such an approximation approach that borrows from variational calculus by introducing a parameterized function q(θ) that will be shaped, through an optimization procedure, to approximate the true posterior q(θ)≈p(θ|y) . This optimization procedure requires the definition of an objective function, which is typically the evidence lower bound (ELBO), sometimes also called the variational lower bound or the free energy (particularly in the context of DCM) due to its resemblance to the functional form of the free energy in physics (Zeidman et al., 2022).

There are different ways to derive the ELBO; one approach is to start with the Kullback-Leibler (KL) divergence between the variational distribution and the posterior $D_{KL}(q\left(\theta \right)||p(\theta |y))$ (Cover & Thomas, 2006). Minimizing $D_{KL}$ is equivalent to maximizing the ELBO:

$$ELBO=\text{log}\left(p\left(y\right)\right)-D_{KL}(q\left(\theta \right)||p(\theta |y))$$ (10)

Note that the KL divergence is only well defined if q has probability zero at the same intervals as p; in other words, the support of the two distributions must be the same.

Applying the Laplace approximation to simplify this problem means assuming a Gaussian variational posterior $q\left(\theta \right)=N\left(\mu_{\theta },\Sigma_{\theta }\right)$. This is the approach taken by DCM (Novelli et al., 2024).

**Fig. 1.**
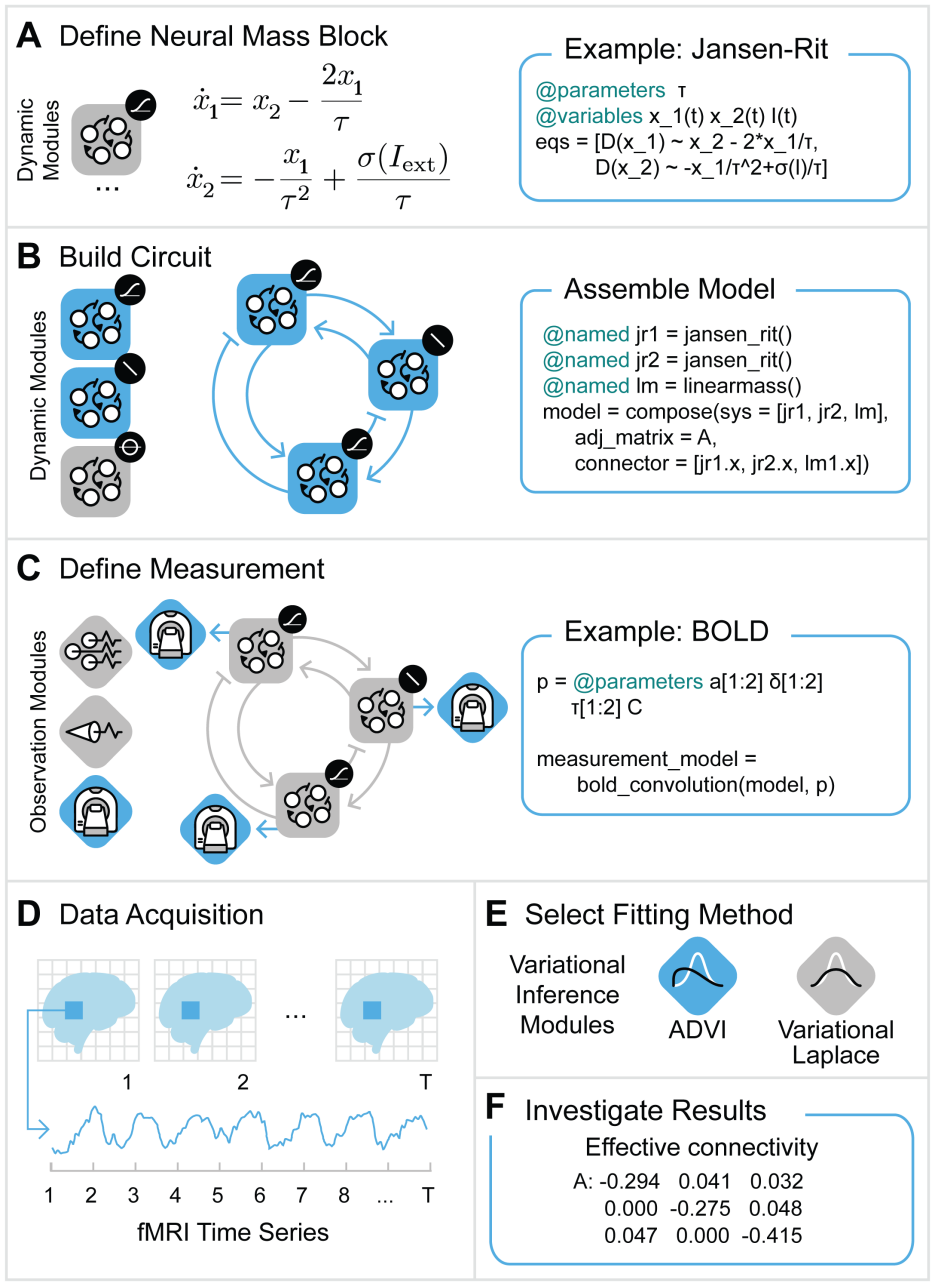
Modular design for user-friendly circuit model assembly and data fitting. Blue boxes on the right side in panels (A) to (C) show code snippets of the model assembly illustrated on the left. Note that the blue icon color illustrates selection in the respective panel and carries no additional meaning. In (A), the definition of a model (also called block) is shown, in this case a Jansen-Rit model. Coding it is almost as simple as writing its differential equations. A user can pick and choose from a library of available models (panel (B) on the very left illustrates a few choices disambiguated by the small black icon on the top-right corner of the large icon). From top to bottom: Jansen-Rit model, linear neural mass model, and next-generation neural mass model. Neuroblox.jl provides most of the common neural mass models. A linear neural mass and two Jansen-Rit blocks are selected and connected to form a circuit. In addition to a model for the neuronal activity, we select (or define a new) measurement modality in (C); in this particular case, we assume fMRI measurements and thus add a BOLD signal model. For LFP modeling, lead-field equations are available in the package. Every region can, in principle, be described by a different neural mass model which substantially expands modeling power over the SPM toolbox, where regions are constrained to have dynamics of the same functional form. Lastly, a fitting procedure can be selected (E) and parameter values estimated (F) from acquired data (D). (Graphic in (D) adopted from CC-licensed graphic in Sidén, 2020).

Here, we present several advances that expand the capabilities of spectral DCM as implemented in SPM. We provide a Julia implementation with a user-friendly interface for adding new models and linking them in arbitrarily complex circuits. Our implementation also includes built-in corrections for different acquisition field strengths not previously considered in DCM analyses. Our software leverages ModelingToolkit.jl, a symbolic computation framework written Julia, to achieve a high degree of modularity and composability (Fig. 1). While the implementation of DCM in Julia already increases computation speed over the traditional MATLAB version, we also employ automatic differentiation to further improve it.

## 3 Methods

### 3.1 Modular design for user-friendly circuit model assembly and data fitting

Defining a model should be as simple as writing down its equations. For this purpose, our toolbox design leverages ModelingToolkit.jl (Ma, Gowda, et al., 2021) and Symbolics.jl (Gowda et al., 2022) for symbolic computation, which provide a simple and intuitive way to define a model that consists of differential equations (see Fig. 1A).

**Fig. 2.**
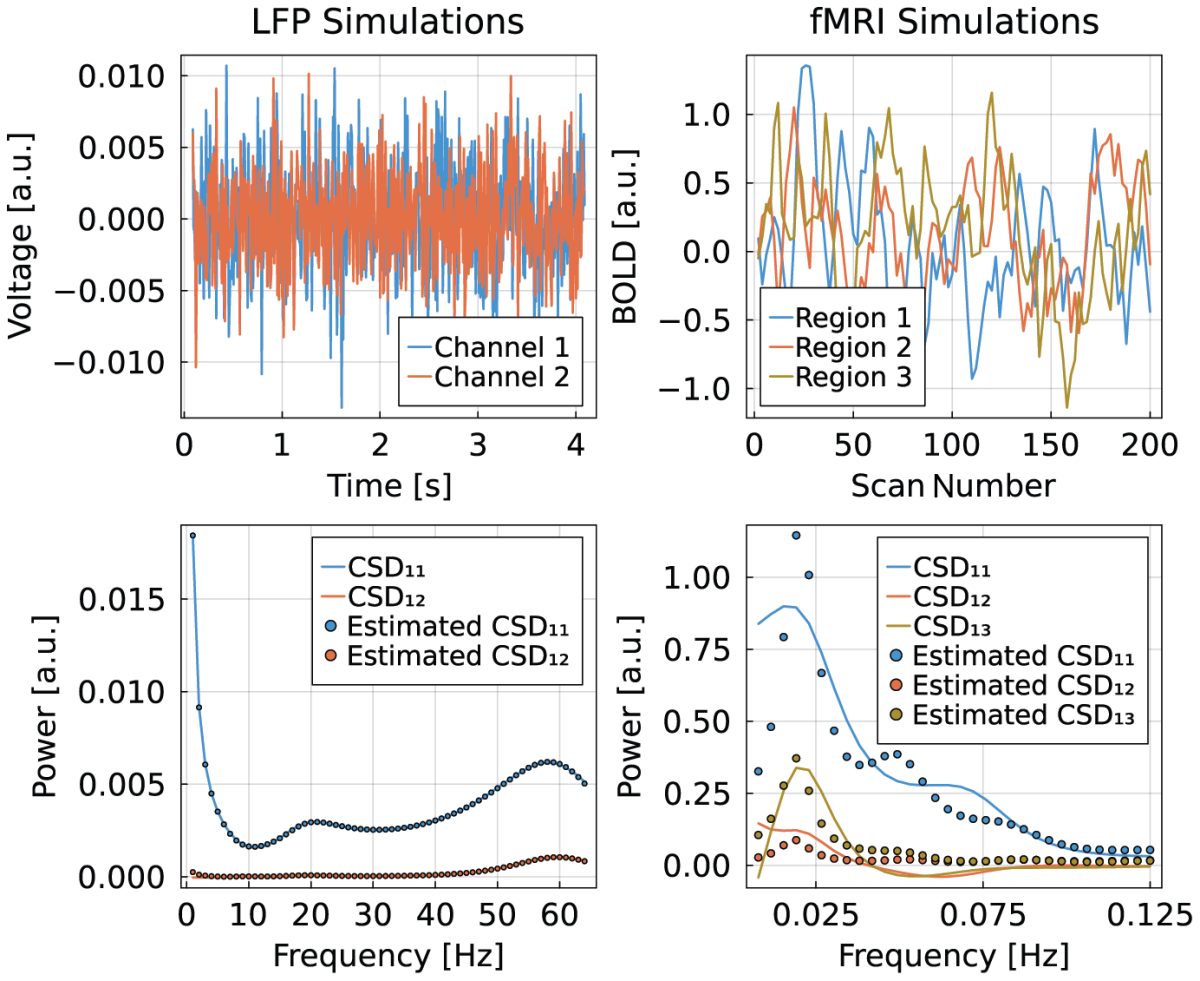
Estimation of cross-spectral densities of simulated data. Top row are examples of simulated signals, left are LFP measurements of two channels, and right are fMRI measurements of three regions. Plots in the bottom row are examples of simulated (lines) and estimated (dots) cross-spectral densities. We find a nearly perfect match for LFP signals, while fMRI signals show only a very rough agreement. The key difference is the nonlinear transformation of the neuronal signals through the hemodynamic response function; the LFP signals are only affected by a constant gain term that may vary for each channel. These simulations are based on the SPM scripts *DEM_demo_induced_FMRI.m* for fMRI signals and *spm_csd_demo.m* for LFP signals. For details about the LFP model, see R. J. Moran, Jung, et al. (2011).

For instance, the user can define arbitrary models describing the dynamics of a particular brain region and assemble them into an overall model through an adjacency matrix (see Fig. 1B). In this paper, we studied two different systems for neuronal dynamics: A simple linear ODE system as is typically used in SPM for the spectral DCM analysis of fMRI signals (K. J. Friston et al., 2014), and a more complex canonical micro-circuit model which is used to model LFP signals (Bastos et al., 2012; R. J. Moran, Jung, et al., 2011; R. J. Moran et al., 2013) (see Fig. 2). The definition of an observation model that relates the (hidden) neuronal activity to the actual neurophysiological measurement completes the model assembly (see Fig. 1C). The user can pick a variational inference algorithm by providing measurements corresponding to the observation modality (Fig. 1D). Besides Variational Laplace that is employed by SPM and studied in this manuscript, we also provide Automatic Differentiation Variational Inference (ADVI) as implemented in the library Turing.jl (see Ge et al., 2018; Kucukelbir et al., 2016) (Fig. 1E) to estimate the posterior probability of the model parameters, for instance the effective connectivity between regions (Fig. 1F).

The toolbox provides a Variational Laplace inference algorithm analogous to the SPM implementation and a Variational Laplace implementation that leverages automatic differentiation for the computation of the model Jacobian, both with and without symbolic computation. The inference is performed on the cross-spectral densities of the measured time series. The initial conditions of the states need to be selected to be in a steady-state solution.

### 3.2 Automatic differentiation

Julia provides different libraries for performing automatic differentiation. We chose ForwardDiff.jl because of its robustness and good performance for systems with up to 100 dimensions (Ma, Dixit, et al., 2021; Revels et al., 2016).

The SPM implementation of the matrix-exponential of the kernel κ(τ) given in Eqn. 6 requires an eigenvalue decomposition. This poses a particular challenge since the eigenvalue decomposition in the standard library LinearAlgebra.jl is not a native Julia implementation but is based on the external, compiled BLAS library and, thereby, is not amenable to automatic differentiation. The package GeneralizedLinearAlgebra.jl provides linear algebra operations that are fully implemented in Julia; however, its eigenvalue decomposition only provides eigenvalues but not the corresponding eigenvectors at the point of writing this manuscript.

Thus, we created a dispatch of the eigenvalue decomposition performed by the function *eigen* defined in LinearAlgebra.jl. Dynamic dispatch is the process of selecting which implementation of a polymorphic operation to call at run time. Polymorphic operation refers to the possibility to define different operations, in our case functions, that carry the same name but perform different operations. A dispatch of a function is a specific implementation of a function with an interface that differs in terms of the number or types of arguments. This allows the programmer to define a particular behavior for particular types, such as the eigenvalue decomposition of a matrix of type Dual, as is our case. The dispatch implements the analytical derivative of eigenvalues and eigenvectors (Lin et al., 2020; Van der Aa et al., 2007). However, the analytic derivative of eigenvectors with degenerate eigenvalues has poles and becomes numerically unstable as these degeneracies are approached. Indeed, the standard SPM initial conditions for the linear model and balloon model, which are by default set to zero, result in a Jacobian with degenerate eigenvalues. To avoid this degeneracy when necessary, we introduce small Gaussian perturbations (of the order $O(10^{-10})$ ) to the initial conditions, as well as to the prior expectation value of the connection matrix. For dynamic models that do not suffer from this problem (e.g., coupled canonical microcircuit), we keep the SPM standard and set all states to 0, that is, to the model’s trivial fixed point.

Since the Dual type of ForwardDiff.jl is not defined for complex numbers, separating the real and imaginary parts in our code base is necessary. This approach improved execution speed over a previous implementation that attempted to address similar problems (Thummerer & Mikelsons, 2023).

### 3.3 Symbolic computation

Using symbolic computation provides two important advantages. First, it facilitates modular definitions of models, making them easily extensible, alterable, and composable. Second, it provides a means of automatic analytical computation of gradients and Jacobians, which avoids the potentially imprecise numerical computation of derivatives or the effort of hard-coding the analytical derivatives for each new model. In spectral DCM, three multidimensional derivatives need to be computed. For the optimization procedure, we need to compute the Jacobian with respect to the model parameters (K. J. Friston et al., 2014; Novelli et al., 2024; Zeidman et al., 2022).

In SPM, this is evaluated by computing the finite difference of the code-based function that involves computing the cross-spectral density Eqn. 8, which depends on a cascade of computations, among others on the calculation of the gradient with respect to the system states of the measurement function g and the Jacobian of f (see Eqn. 6). This suggests that evaluating this Jacobian with finite differences can be computationally costly and potentially inaccurate (see Baydin et al. (2018)). Hence, this Jacobian is a formidable candidate to be computed by automatic differentiation, which only requires a single function evaluation. Indeed, as shown in the results section, we achieved a substantial speed improvement due to automatic differentiation.

Symbolic computation is implemented by packages ModlingToolkit.jl and Symbolics.jl (Gowda et al., 2022; Ma, Gowda, et al., 2021)

## 4 Simulation Results

### 4.1 Assessment of validity of the Julia implementation

We perform a series of tests on simulated toy data to ensure that our Julia implementation of spectral Dynamic Causal Modeling provides the same results as the SPM implementation. We studied two different systems: a simple linear ODE system along with a biophysical model for the hemodynamic response as is typically used in SPM for fMRI signals, and a more complex canonical micro-circuit model with lead field potentials as measurement function, which is used to model LFP signal.

The hemodynamic and BOLD signal model is explained in detail in this manuscript in equations Eqn. 2 to Eqn. 5. The lead field potential (LFP) is modeled by weighting the output of the neural mass model; typically, the pyramidal neurons of the CMC are considered to produce the signal that is being measured. Accordingly, the output neuronal mass is the superficial pyramidal neurons.

Our results based on simulations of linear neural mass models as well as CMC simulations demonstrate near-identical parameter estimates for our novel Julia implementation and the existing MATLAB implementation (SPM25; Table 1). Note the parameter deviation for the CMC circuit while the estimation of effective connection strengths for the linear model with nonlinear HRF is substantially closer to the ground truth.

**Table 1.** Comparison of estimated parameter expectations between SPM25 and our Julia implementation for simulations of the linear neural mass model, which is typically used to model fMRI signals, based on the SPM script *DEM_demo_induced_fMRI.m* and the canonical micro-circuit, based on the SPM script *spm_csd_demo.m*.

fMRI	SPM25	Julia	ground truth	CMC	SPM25	Julia	ground truth
$a_{12}$	-0.1969	-0.1969	-0.2	$a_{dp\to sp}$	0.4181	0.4181	1.0645
$a_{21}$	0.3348	0.3348	0.4	$a_{dp\to ii}$	10.7195	10.7195	20.0855
$a_{32}$	-0.2863	-0.2863	-0.3	$a_{sp\to ss}$	0.9237	0.9237	1.6487
$a_{23}$	0.1996	0.1996	0.2	$a_{sp\to dp}$	1.1089	1.1089	4.4817
$\tau_{1}$	0.8858	0.8857	1.2499	$L_{1}$	0.9797	0.9799	1
$\tau_{2}$	0.9303	0.9303	1.2374	$L_{2}$	0.8718	0.8719	1
$\tau_{3}$	1.0234	1.0234	1.0743	r	1.0567	1.0567	1
F	-400.5302	-400.5289	-	F	1817.1634	1817.1660	-

Here, $a_{x}$ are effective connection strengths, $\tau_{x}$ are the logarithm of transient times of the hemodynamic model, F is the free energy, $L_{1/2}$ are the lead field factors, and r is the slope parameter of the sigmoidal function in the Jansen-Rit model.

Table 1 reveals an important limitation: estimated parameters sometimes deviate from their true values, particularly in the CMC example. This is a common issue in Bayesian parameter estimation. The systematic underestimation we observe likely stems from the zero-centered prior distribution. While a thorough prior sensitivity analysis would be valuable, it falls outside this article’s scope. This highlights a broader concern: DCM’s choice of Gaussian priors can significantly influence both parameter estimation and model selection, as Litvak et al. (2019) demonstrated in their detailed analysis.

### 4.2 Julia implementation provides substantial speed improvements

Computational complexity can pose a bottleneck when analyzing high-dimensional datasets covering multiple brain regions. Julia provides a high-performance computing environment that reaches computational speeds comparable to C/C++ due to just-in-time compilation (Bezanson et al., 2018; Pelenitsyn et al., 2021). We implemented the variational inference method based on the Laplace approximation, also called Variational Laplace, for parameter estimation analogous to the implementation in SPM. We compare our method based on simulated toy data with the SPM25 implementation to ensure consistency and correctness of our implementation in Julia.

**Fig. 3.**
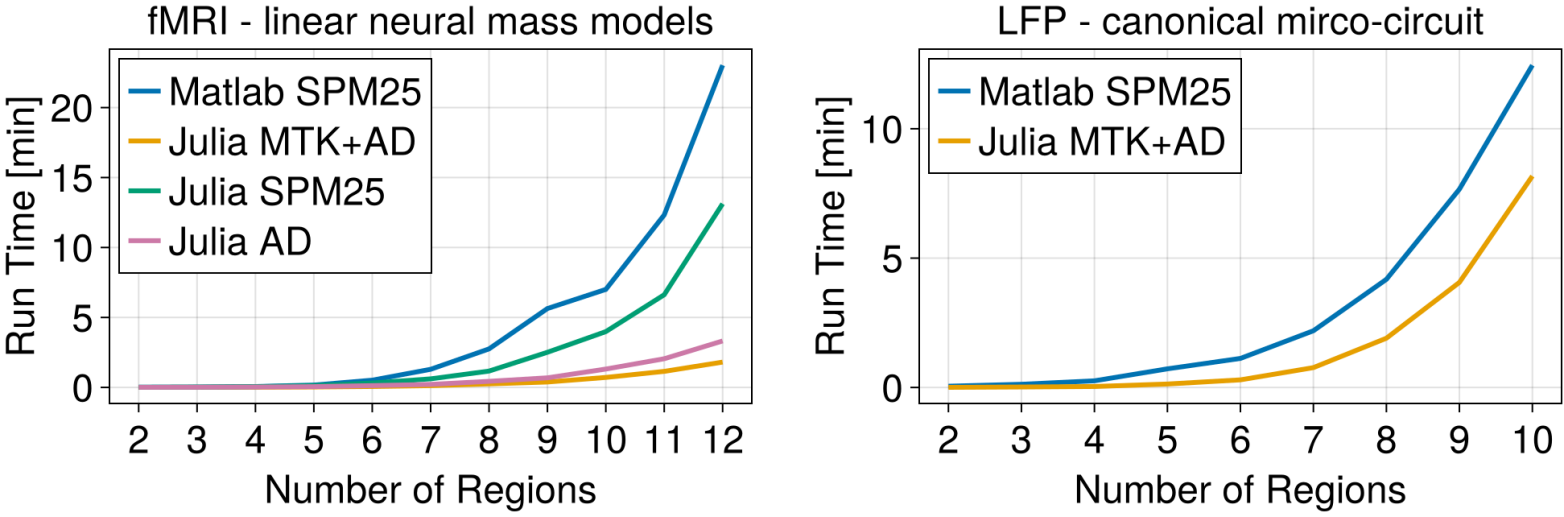
Julia implementation provides substantial speed improvements. Graphs show the speed increase compared to the MATLAB SPM (blue) implementation as a function of the number of regions. The left graph compares different DCM implementations on simulations of fMRI data. As typical DCM is based on a linear neural mass model. Notable speed improvement is already achieved by a plain Julia re-implementation of the SPM DCM (green). Employing Automatic Differentiation (AD) results in an additional speed improvement (rose) and by adding symbolic computation speed increases further by a small factor to reach an increase of more than 10-fold, as observed by the orange line. The right graph shows the speed comparison for a more complex model such as the CMC. Here, the speed gain is substantially more tempered, giving a 1.5 (10 regions) to 2-fold (8 regions) improvement.

We find that the Julia implementation provides a meaningful speed improvement (Fig. 3) while yielding the same results in terms of posterior means and free energy for all the different implemented versions.

These results were achieved using simulated data from the SPM script *DEM_demo_induced_FMRI.m*. However, we note that when fitting coupled canonical microcircuits to simulated LFP data, we find that minimal deviations (of order $O(10^{-3})$ to $O(10^{-4})$ ) in parameter expectation value and free energy between the SPM implementation and the Julia version that makes use of AD and symbolic computation can occur (see Table 1). The simulation for this analysis is performed using the script *spm_csd_demo.m*.

Note that in SPM we need to set the DCM option “maxnodes” to a value equal to or larger than the number of inferred regions to ensure a correct comparison. Maxnodes will otherwise reduce the problem’s dimensionality to the value provided, leading to a lower computational load.

Finally, we made the code amenable to automatic differentiation and used Julia’s ForwardDiff.jl library to perform automatic differentiation on the Variational Laplace optimization. We find a further speed improvement, as can be observed in Figure 3, yielding an almost order of magnitude speed improvement for 10 regions over the MATLAB SPM implementation.

### 4.3 Bayesian model comparison

In Dynamic Causal Modeling, parameter fitting is typically the first step, followed by model selection. While various methods exist for model selection, a standard approach in DCM is to compare the free energy of different models (Novelli et al., 2024; Zeidman et al., 2022). We performed this type of model selection to demonstrate consistency between the Julia and MATLAB implementations. However, it is worth noting that more modern methods, such as Parametric Empirical Bayes, have been proposed to address some limitations of the standard approach (Zeidman et al., 2019).

In the standard SPM approach, prior distributions are adjusted to include or exclude parameters by setting the parameter’s variance and mean to zero. This effectively removes the parameter from the model, preventing it from being modified during the optimization process (zero variance). In our Julia implementation, parameters are included or excluded in the optimization procedure using a *tunable* flag. Setting the *tunable* flag of a symbolic parameter to *false* excludes it from optimization, though it does not automatically set its mean to zero. This allows a parameter to retain a fixed, non-zero mean during optimization, representing a fixed effective connection that remains unchanged. For all the examples reported in this study, the default behavior of setting effective connections to zero, as in SPM, is maintained.

As described in the previous section, we compared models using synthetic data generated by simulating a standard linear neural mass model with the balloon model. These simulations were performed using the SPM script *DEM_demo_induced_fMRI.m*. Model comparison was based on the negative free energy values (see Table 2). The results show that the ground truth model outperforms all other models except for one of lower complexity, where the effective connection between regions 2 and 3 was removed. This simpler model exhibited a free energy value that was insignificantly different from the ground truth model. In Table 2, we report instances where the free energy values obtained from SPM differ from those obtained in the Julia implementation. These discrepancies are rare and statistically insignificant, leading to identical conclusions regarding model selection.

**Table 2.** Model comparison is performed by comparing free energies after removing or adding different parameters to the prior distribution.

$a_{12}$	$a_{21}$	$a_{23}$	$a_{32}$	$a_{13}$	$a_{31}$	Relative F
1	1	1	1	0	0	0
0	1	1	1	1	0	-3.34
1	0	1	1	1	0	-145.25
1	1	0	1	1	0	-2.69
1	1	1	0	1	0	-53.13
0	1	1	1	0	1	-3.23
1	0	1	1	0	1	-149.44
1	1	0	1	0	1	-2.19
1	1	1	0	0	1	-40.85 (-40.86)
0	1	1	1	0	0	-1.1
1	0	1	1	0	0	-149.02 (-149.03)
1	1	0	1	0	0	-0.35
1	1	1	0	0	0	-50.71
0	1	1	1	1	1	-4.85
1	0	1	1	1	1	-157.53 (-157.54)
1	1	0	1	1	1	-4.48
1	1	1	0	1	1	-49.26 (-49.27)
1	1	1	1	0	1	-2.39
1	1	1	1	1	0	-2.56
1	1	1	1	1	1	-4.66 (-4.67)

Here, we show relative F for different models, that is, the free energy difference between the specific model and ground-truth model $F-F_{0}$.In SPM, when a parameter is removed from the model its variance and mean are set to zero. We perform model comparison accordingly, thus a 0 in the table means that the specific parameter is absent and mean and variance are set to zero. A value of 1 implies a variance of 1/128 while the mean is still initiated with 0. $a_{xy}$ denotes the effective influence exerted by region x onto region y. The top part of the table shows models of the same complexity, that is, number of parameters, as the ground-truth model (first row of the table), the middle part shows the under-complex model, and the bottom part over-complex models. Free energy F is maximized. Therefore, the best model is the one with the largest (least negative) free energy. Generally, the results are attained with the implementation based on automatic differentiation. We add the results attained from the plain implementation in brackets whenever we identify a deviation between the values of at least the second floating point digit as compared to the AD version. The toy data is created using the fMRI model simulation given in the SPM script *DEM_demo_induced_fMRI.m*.

## 5 Experimental Data Example

### 5.1 fMRI pre-processing methods

As we are validating our pipeline against spectral DCM (K. J. Friston et al., 2014), we use a sample subject from the FC1000 project (available at www.fil.ion.ucl.ac.uk/spm/data/spDCM/)—one of the group used by K. J. Friston et al. (2014) in their initial validation of spectral DCM—to demonstrate the coherence between SPM and Neuroblox implementations of DCM. The scan was acquired at the University of Oxford Centre for Clinical Magnetic Resonance Research using a 3T Siemens scanner. Whole-brain fMRI was acquired using a gradient-echo EPI sequence (Repetition Time ($T_{R}$) = 2000 ms, Echo Time ($T_{E}$) = 28 ms, flip angle = 89,° field of view = 224 mm, voxel size 3 mm x 3 mm x 3.5 mm, 180 volumes acquired). This scan was accompanied by a T1-weigheted structural MRI ($T_{R}$ = 2040 ms, $T_{E}$ = 4.7 ms, flip angle = 8°, field of view = 192 mm, voxel size = 1 mm x 1 mm x 1 mm) to provide tissue segmentation. The fMRI scan was preprocessed by removing the first five volumes, realigning to the reference scan, normalizing to MNI space, correcting for slice-timing, and spatially smoothing the data with a Gaussian kernel (6 mm FWHM). A GLM containing movement regressors was used to remove confounds, along with confound GLMs fit from the ventricle (CSF) and white matter signals (see SPM manual for details). Four ROIs were selected from the default mode network (DMN); all were chosen as 8 mm radius spheres centered in each location. The four regions are the posterior cingulate cortex (PCC; center [0, -52, 26]), medial prefrontal cortex (mPFC; [3, 54, -2]), left intraparietal cortex (LIPC; [-50, -63, 32]), and right intraparietal cortex (RIPC; [48, -69, 35]).

### 5.2 Comparison of SPM and Julia DCM fitting procedures on fMRI data

The DCM was fitted using the latest SPM version (25), following a standard pipeline described above and in the SPM manual to ensure reproducibility. The Julia implementation accurately reproduces the same results as the SPM version, thus we report the effective connections extracted from empirical data of both in a single table (Table 3).

**Table 3.** We report effective connection strengths estimated between several brain regions.

Julia/SPM25	mPFC	PCC	RIPC	LIPC
mPFC	0.21	0.014	0.11	0.34
PCC	0.16	0.45	0.29	-0.08
RIPC	0.53	0.11	0.04	0.09
LIPC	-0.21	0.25	0.21	0.27

Note that the Julia implementation (indeed all three versions) provides the same results, thus we do not present it in a separate table. The comparison is performed using empirical data, which can be found at https://www.fil.ion.ucl.ac.uk/spm/data/spDCM/.

In this empirical example, we fit a fully connected DCM (i.e., the four regions are all-to-all connected) with no exogenous inputs specified and using the default parameter values (sequential data, $T_{E}=0.04$ , no modulatory effects, one state per region, no stochastic effects, no centering of input). Furthermore, we probed the effects of field strength correction on the results by fitting at three different field strengths (1.5T, 3T, and 7T), presented in the Supplementary Materials (see Supplementary Table S2). Each field strength yielded significantly different results than the other. Still, all fits agreed in the directions of inter-regional connections, except for LIPC intraregional connectivity (reversed at 1.5T).

## 6 Discussion

DCM is one of the most widely used and validated methods to extract underlying neural connectivity from fMRI data by fitting and selecting models (Cha et al., 2016; R. J. Moran, Mallet, et al., 2011; Novelli et al., 2024; Rupprechter et al., 2020; Seghier & Friston, 2013; Tik et al., 2018; Zarghami et al., 2023). The aim of the work presented here is two-fold: to improve the computational speed and accessibility of the well-established spectral DCM framework provided by SPM (K. J. Friston et al., 2014), and to increase the application domain by providing a modular framework for model fitting, thus empowering researchers to tailor their models to specific experimental setups and research questions. Importantly, each brain region can be defined as governed by a different system of differential equations, which provides more flexibility than SPM, where all regions are governed by the same system of differential equations (with potentially different parameters). We demonstrate that our implementation offers up to an order of magnitude improvement in computational speed compared to the SPM25 DCM, an enhancement that enables efficient analysis of large-scale datasets and complex models.

Our work builds upon spectral DCM by re-implementing it in Julia, a high-performance programming language (Ma, Dixit, et al., 2021; Rackauckas, 2023). By implementing the SPM DCM variational Laplace approach in Julia, we provide a method that recovers identical connectivity values in both simulated and human fMRI data, while significantly reducing computation time. Although SPM is distributed using an open-source license, it relies on MATLAB, which requires a commercial software license. Julia, on the other hand, is a fully open-source (MIT license) with a specific focus on scientific computation (Bezanson et al., 2018; Danford et al., 2017; Freeman, 2015; Huang et al., 2019; Kramer et al., 2018; Ma, Dixit, et al., 2021). Julia is a just-in-time compiled language designed for high-performance computing, resulting in an order-of-magnitude increase in execution speed over similar MATLAB implementations (Danford et al., 2017; Rackauckas, 2023). We further optimized spDCM by employing automatic differentiation (Revels et al., 2016), which can improve the accuracy of results for complex non-linear models (Griewank & Walther, 2008).

A limitation of standard DCM implementation is that all but the most technically savvy users who feel comfortable editing the base code are constrained to the modeling choices imposed by the software. These prespecified models are a linear neural mass (attached to a balloon model for fMRI fitting (K. J. Friston et al., 2014)) or a few varieties of neuronal mass and neural field models to fit LFP and EEG recordings (R. J. Moran, Jung, et al., 2011; R. J. Moran et al., 2013). In our Julia spDCM implementation, we allow the user to create their own dynamic models in ModelingToolkit (Ma, Gowda, et al., 2021) or Neuroblox (Neuroblox.org) (see, for example, the Janson-Rit model in Fig. 1A).

To illustrate the utility of a flexible modular approach, we provide a method to improve correction for MRI scanner field strengths when fitting a model to real data provided in the Supplementary Materials. Given the ubiquity and power of DCM approaches, it is necessary to consider advances in MRI scanner technology that have happened since the initial implementations of DCM, when 1.5T and 3T scanners were predominately used for fMRI (K. J. Friston et al., 2014; Stephan et al., 2008). Here, we demonstrate how accounting for higher field strengths (e.g., 7T) that have become more common in human neuroimaging studies is an important consideration, as the results of fitting with the wrong field strength can yield significantly different results. We note that the directionality of inter-regional connections is largely stable to this field strength correction (i.e., network topology does not change although the connection strengths do), giving us confidence that prior studies using this technique are still valid. However, given the noticeably different magnitudes of effective connectivity strengths and the fact that this metric is used in many studies as the outcome of interest, it is important to apply the field strength of the original data when using spDCM. We provide that capability in the package presented here.

More recently, regression DCM was introduced, providing a powerful technique in terms of speed and accuracy (Frässle et al., 2017, 2021). As spectral DCM, regression DCM does require the cross-spectral density to be stationary. While regression DCM demonstrated superior accuracy compared to spectral DCM (Frässle et al., 2021), it currently has a significant limitation: the method is restricted to linear models and cannot incorporate biologically realistic neural mass models, such as the canonical micro-circuit. While recent evidence suggests that linear models are sufficient models for fMRI data in resting state with current measurement resolution constraints (Nozari et al., 2024), there are several scenarios where linear models may not suffice (Bastos et al., 2015; K. J. Friston et al., 2019).

Our optimization implementations represent initial steps toward advancing neural modeling techniques. The open-source nature of our framework specifically facilitates future development and optimization. Alternative approaches could enhance this foundation, including the use of different variational optimization methods such as ADVI (Kucukelbir et al., 2016) or the NUTS sampler (Hoffman & Gelman, 2014), both available through Turing.jl (Ge et al., 2018). By leveraging the Julia ecosystem, our method provides a flexible platform to accelerate the development of neural models at the level of brain regions.

## Supplementary Material

Supplementary Material

## Data Availability

All methods shown here, including documentation and tutorials, are freely available through the Neuroblox computational neuroscience platform (Neuroblox.org). Neuroblox.jl can be accessed through Github: github.com/Neuroblox/Neuroblox.jl. The Julia code base is available here: github.com/Neuroblox/Spectral-Dynamic-Causal-Modeling.git
