# Supplementary material for: Increasing spectral DCM flexibility and speed by leveraging Julia’s ModelingToolkit and automated differentiation
Source: Imaging Neurosci (Camb). 2025 Jul 24;3:IMAG.a.88. doi: 10.1162/IMAG.a.88 (PMC12330849; doi:10.1162/IMAG.a.88)
Supplement: Supplementary Material [file IMAG.a.88_supp.pdf]

A Supplemental Materials

To further illustrate the flexibility of the implementation presented here, we highlight the importance of being able to fit fMRI data at different field strengths to match the field of acquisition. While this is possible in the traditional SPM implementation, it requires modifying the base code should field strengths other than the preset options be desired. As we illustrate in this supplemental materials, the field strength used to fit the data can significantly impact the results of the fit, and is therefore an important parameter for users to have easily accessible to best fit their data.

Influence of field strength on hemodynamic variables in Balloon-Windkessel model

In the hemodynamic model DCM uses, six parameters can affect the fit substantially. Five of them have been discussed at length elsewhere (Buxton, 2013; Friston et al., 2003; Obata et al., 2004), and so will be briefly restated here. The echo time (T_E) is always taken directly from the data being fit and is thus specified by individual users (Friston et al., 2014). The resting venous volume percent (V_0) and resting oxygen extraction fraction (E_0) are physiological parameters that - while variable across individuals - do not vary systematically in different field strengths and are thus assumed to be single values established in prior work (Buxton, 2013; Friston et al., 2003; Obata et al., 2004). The ratio of intra- to extravascular signal (ϵ) is fit on a region-by-region basis, which has been shown to improve accuracy (Stephan et al., 2007). The slope of intravascular relaxation rate as a function of oxygen (r_0) can potentially vary at different field strengths; however, at fields above 3T, the contribution of this term becomes negligible due to the dominance of k_1 in the balloon model (see supplemental materials and in (Obata et al., 2004) for a full discussion of this effect). Given prior work that has demonstrated improved accuracy varying this parameter (Havlicek et al., 2015), however, we also vary it with ν_0, as described below.

The remaining parameter of the balloon model is the maximum frequency offset at the outer surface of a magnetized vessel (ν_0). This value has been shown to vary in different field strengths, and the exact derivation of this quantity has been addressed at length elsewhere. Summarizing this work (see (Buxton, 2013)): the maximum frequency offset at the surface of a blood vessel depends on the gyromagnetic ratio of protons in a field ($\gamma \approx 128$MHz), the susceptibility difference ($\Delta\chi_0 = 0.264 \times 10^{-6}$), the hematocrit concentration ($H = 0.44$), the venous saturation ($Y = 0.6$), and the field strength B_0, yielding the final expression of frequency offset

$$\nu_0 = \gamma \Delta\chi_0 H(1 - Y)B_0$$

Prior work has estimated different values depending on different assumptions of hematocrit, venous saturation, or susceptibility difference, and individual variation (e.g., sex differences in baseline hematocrit concentration (Buxton, 2013)) can cause significant variability of this constant. However, for the work at hand, we note the linear dependence on field strength, with a doubling of this constant when moving from 1.5T to 3T, and even greater differences when moving to ultra-high fields (7T and 9.4T) used in modern human imaging studies. We probe these differences further in simulations and real data. In the following sections, the values for ν_0 and r_0 at different field strengths are taken from prior work (see (Havlicek et al., 2015) Tab. 1.B.). We do not vary the prior on ϵ purely for the sake of these illustrative examples, but there is no constraint on the user preventing them from doing so.

Influence of field strength on hemodynamic response function

While there are many possible reasons for the effect that varying ν_0 with field strength has on the fitting results of DCM, one important consideration is a direct effect on the shape of the hemodynamic response function (HRF). In this section, we probe the effect of field strength on the Balloon-Windkessel model HRF to determine what, if any, these effects are.

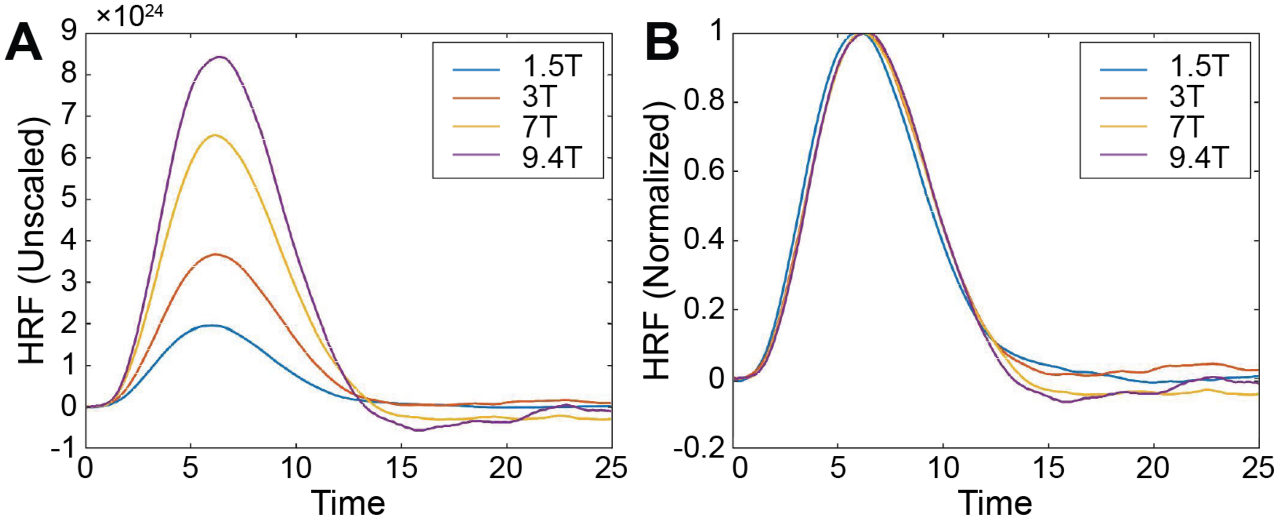

Figure S1: **Field strength affects magnitude and shape of hemodynamic response function in Balloon-Windkessel model.** A. The primary effect of field strength (captured by ν_0) is the increase in magnitude of the HRF. The HRFs plotted here are the result of integrating the HRF in response to a Dirac delta pulse (simulated at TR=0.5s). B. To examine more subtle effects which remain after normalizing the fMRI signal, all field strengths from A are plotted on a normalized scale.

First, we must compute the HRF from the balloon model. As there are potential differences due to numerical solver effects between noisy simulations and pulsed simulations, we provide examples of both illustrating the effects of the varying field strength on the HRF. First, we compute the HRF in response to a Dirac delta pulse applied for a single TR. The results of these computations are shown in Fig. S1.

Fig. S1A shows the strongest effect of field strength on the HRF - namely, the increase in magnitude of the response curve. However, as this can be removed by standard fMRI pre-processing techniques (signal normalization), Fig. S1B shows the same HRF curves normalized to a common scale. In this case, we observe a slight delay ($\sim$250ms) in the peak times of higher fields (3T, 7T, and 9.4T) compared to the lowest field (1.5T). More importantly, the characteristic undershoot of the HRF takes on different depths depending on the field strength. Finally, it appears that the overall width of the peaks does not vary significantly.

To further probe this variability, we simulated the HRF model 50x at four different field strengths (1.5T, 3T, 7T, and 9.4T) common in human neuroimaging studies. Rather than using a simulated neuronal signal, we input white noise into the model to allow for computing the HRF directly from simulated data via division in the Fourier domain.

Considering the original neuronal signal $x(t)$ and the output BOLD signal $y(t)$, their relationship can be described by a convolution with an HRF $h(t)$ such that

$$(x * h)(t) = y(t)$$

Converting this to the Fourier domain, this becomes a simple multiplication problem

$$x(\omega)h(\omega) = y(\omega)$$

and the HRF can be determined by simple division if the input signal $x(t)$ is white noise (guaranteeing $x(\omega)$ is a constant). We note that this approach does not work in most cases, as real signals can easily produce zero values in the Fourier domain, leading to a division by zero. However, in the case of pure noise as is considered here, we know the Fourier transform to be nearly constant with only minor variability, and so this approach is justified.

To further quantify the HRF differences across field strengths, we computed the mean peak and full width at half of the peak of each signal (results are shown in Tab. S1). We note that although there is a

	1.5T	3T	7T	9.4T
Time to peak (ms)	6023 (506)	6235 (390)	6180 (476)	6223 (575)
Width at half peak (ms)	6323 (1091)	6480 (1091)	6283 (591)	6289 (698)

Table S1: HRF characteristics at varied field strengths.

slight delay in the average time-to-peak of higher fields compared with 1.5T, this lies comfortably within the variance between simulations, and does not therefore make a strong contribution to variance in fits at different field strengths. Likewise, the duration of the HRF does not vary significantly across field

strengths. This leads us to conclude that if the shape of the HRF does indeed play a role in goodness of DCM fits, it is in the more accurate representation of HRF undershoot at higher field strengths. We note, however, that there are still many questions regarding optimal fitting of the HRF at different field strengths that are left to a more complete discussion in future work.

Influence of field strength on hemodynamic response function

As field strength can impact the shape of the HRF, and can potentially impact model fitting as a consequence, we fit the same fMRI data presented in the previous section at three different field strengths: 1.5T, 3T, and 7T. As in the prior section, field strength was varied by varying ν_0 and r_0 according to prior work (Havlicek et al., 2015). The results of these fits are presented in Tab. S2 and visualized in Fig. S2.

**1.5T**	PCC	mPFC	LIPC	RIPC
PCC	0.42 (0.088)	0.19 (0.085)	0.24 (0.059)	-0.03 (0.083)
mPFC	0.05 (0.077)	0.13 (0.094)	0.06 (0.053)	0.35 (0.070)
LIPC	0.16 (0.080)	0.56 (0.076)	0.08 (0.087)	0.05 (0.075)
RIPC	0.19 (0.085)	-0.21 (0.084)	0.23 (0.059)	0.24 (0.081)
**3T**	PCC	mPFC	LIPC	RIPC
PCC	0.58 (0.083)	0.11 (0.092)	0.34 (0.062)	-0.01 (0.090)
mPFC	0.10 (0.087)	0.50 (0.084)	0.24 (0.060)	0.18 (0.084)
LIPC	0.11 (0.069)	0.36 (0.069)	-0.01 (0.086)	0.16 (0.070)
RIPC	0.17 (0.089)	-0.03 (0.091)	0.20 (0.063)	0.50 (0.078)
**7T**	PCC	mPFC	LIPC	RIPC
PCC	0.84 (0.075)	0.14 (0.093)	0.39 (0.065)	0.09 (0.094)
mPFC	0.15 (0.091)	0.81 (0.076)	0.35 (0.065)	0.11 (0.092)
LIPC	0.17 (0.075)	0.30 (0.076)	0.20 (0.082)	0.15 (0.080)
RIPC	0.12 (0.093)	0.09 (0.093)	0.26 (0.066)	0.82 (0.073)

Table S2: Fitting DCM from 3T rs-fMRI yields different results across field strengths. Results are presented as DCM fit (standard deviation).

To assess statistical significance of the differences across field strengths, we observe that the outputs of the DCM fits are Gaussian posterior distributions, and the difference between the fits can be computed as the difference of Gaussians where the mean is given as $\mu_{\text{diff}} = |\mu_1 - \mu_2|$ and the standard deviation as $\sigma_{\text{diff}} = \sqrt{\sigma_1^2 + \sigma_2^2}$. Using this definition, we assess statistically significant differences as those whose difference distribution has a cumulative density function < 0.005 at 0 (this is equivalent a one-tailed t-test $p < 0.005$, or two-tailed t-test $p < 0.01$). Turning to the results, we first note that the fits are significantly different across field strengths, with the variance of fit connection weights more than an order of magnitude smaller than the parameters themselves. Second, we note that these associations are

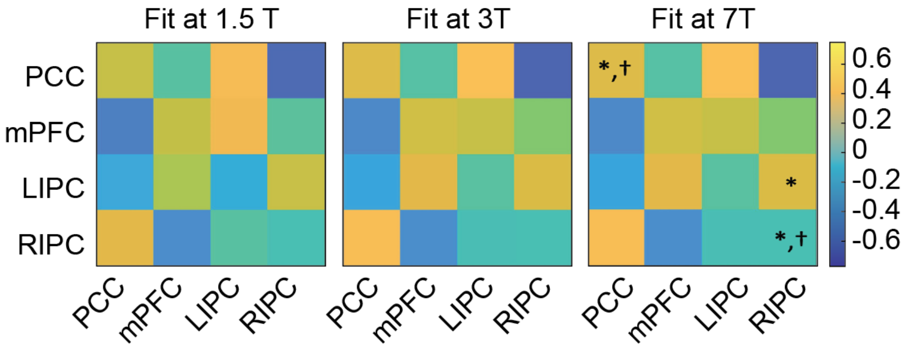

Figure S2: **Visualization of connectivity strengths fit at different HRF field strength corrections.** *Indicates significantly different than 1.5T fit. †Indicates significantly different than 3T fit. It is important to note that while the direction of all fits is the same in most cases, there are more differences in connectivity strengths, particularly noticeable at 7T, the current state of the art for clinical neuroimaging. These differences are especially pronounced compared to the fits done at 1.5T.

not simple linear multiples based on the field strength but significantly impact the relative weights of the associations. For example, mPFC $\rightarrow$ LIPC estimated connection strength increases fourfold when fit at 3T compared to 1.5T (0.06 $\rightarrow$ 0.24), but increases more modestly when fit at 7T compared to 3T (0.24 $\rightarrow$ 0.35). Finally, we note that although these results are significantly different, when examining modern field strengths (3T and 7T), most directions of associations are the same (i.e., the signs of all connection weights agree), although more sign agreement is present between 1.5T and 3T than between 7T and either lower field strength. Thus, while fitting 7T data using 3T parameters is suboptimal, this approach will likely yield directionally accurate results, even if the relative weights are suboptimal. Taking these results together, we believe there is a compelling case that field strength should be accounted for when fitting fMRI data to provide the most accurate results.

References

Buxton, R. B. (2013). The physics of functional magnetic resonance imaging (fMRI). *Reports on progress in physics. Physical Society (Great Britain)*, *76*(9), 096601. https://doi.org/10.1088/0034-4885/76/9/096601

Friston, K. J., Harrison, L., & Penny, W. (2003). Dynamic causal modelling. *NeuroImage*, *19*(4), 1273–1302. https://doi.org/10.1016/S1053-8119(03)00202-7

Friston, K. J., Kahan, J., Biswal, B., & Razi, A. (2014). A DCM for resting state fMRI. *NeuroImage*, *94*, 396–407. https://doi.org/10.1016/j.neuroimage.2013.12.009

Havlicek, M., Roebroeck, A., Friston, K., Gardumi, A., Ivanov, D., & Uludag, K. (2015). Physiologically informed dynamic causal modeling of fMRI data. *NeuroImage*, *122*, 355–372. https://doi.org/10.1016/j.neuroimage.2015.07.078

Obata, T., Liu, T. T., Miller, K. L., Luh, W. M., Wong, E. C., Frank, L. R., & Buxton, R. B. (2004). Discrepancies between BOLD and flow dynamics in primary and supplementary motor areas: Application of the balloon model to the interpretation of BOLD transients. *NeuroImage*, *21*(1), 144–153. https://doi.org/10.1016/j.neuroimage.2003.08.040

Stephan, K. E., Weiskopf, N., Drysdale, P. M., Robinson, P. A., & Friston, K. J. (2007). Comparing hemodynamic models with DCM. *NeuroImage*, *38*(3), 387–401. https://doi.org/10.1016/j.neuroimage.2007.07.040
